# Energy-Efficient Respiratory Anomaly Detection in Premature Newborn Infants

**DOI:** 10.3390/electronics11050682

**Published:** 2022-02-23

**Authors:** Ankita Paul, Md. Abu Saleh Tajin, Anup Das, William M. Mongan, Kapil R. Dandekar

**Affiliations:** 1 Department of Electrical and Computer Engineering, Drexel University College of Engineering, Philadelphia, PA 19104, USA; 2 Department of Mathematics and Computer Science, Ursinus College, Collegeville, PA 19426, USA

**Keywords:** wearable, respiratory classification, deep learning, spiking neural network (SNN)

## Abstract

Precise monitoring of respiratory rate in premature newborn infants is essential to initiating medical interventions as required. Wired technologies can be invasive and obtrusive to the patients. We propose a deep-learning-enabled wearable monitoring system for premature newborn infants, where respiratory cessation is predicted using signals that are collected wirelessly from a non-invasive wearable Bellypatch put on the infant’s body. We propose a five-stage design pipeline involving data collection and labeling, feature scaling, deep learning model selection with hyperparameter tuning, model training and validation, and model testing and deployment. The model used is a 1-D convolutional neural network (1DCNN) architecture with one convolution layer, one pooling layer, and three fully-connected layers, achieving 97.15% classification accuracy. To address the energy limitations of wearable processing, several quantization techniques are explored, and their performance and energy consumption are analyzed for the respiratory classification task. Results demonstrate a reduction of energy footprints and model storage overhead with a considerable degradation of the classification accuracy, meaning that quantization and other model compression techniques are not the best solution for respiratory classification problem on wearable devices. To improve accuracy while reducing the energy consumption, we propose a novel spiking neural network (SNN)-based respiratory classification solution, which can be implemented on event-driven neuromorphic hardware platforms. To this end, we propose an approach to convert the analog operations of our baseline trained 1DCNN to their spiking equivalent. We perform a design-space exploration using the parameters of the converted SNN to generate inference solutions having different accuracy and energy footprints. We select a solution that achieves an accuracy of 93.33% with 18× lower energy compared to the baseline 1DCNN model. Additionally, the proposed SNN solution achieves similar accuracy as the quantized model with a 4× lower energy.

## 1. Introduction

A premature newborn infant is one who is born more than three weeks before the estimated due date. Common health problems of these infants include Apnea of Prematurity (AOP), which is a pause in breathing for 15 to 20 s or more [1], and Neonatal Respiratory Distress Syndrome (NRDS), which is shallow breathing and a sharp pulling in of the chest below and between the ribs with each breath [2]. Precise respiratory monitoring is often necessary to detect AOP and NRDS in premature newborn infants and initiate medical interventions as required [3]. Wired monitoring techniques are invasive and can be obtrusive to the patient. Therefore, non-invasive respiratory monitoring techniques are recommended by pediatricians to increase the comfort of infants and facilitate continuous home monitoring [4].

**Figure 1.**
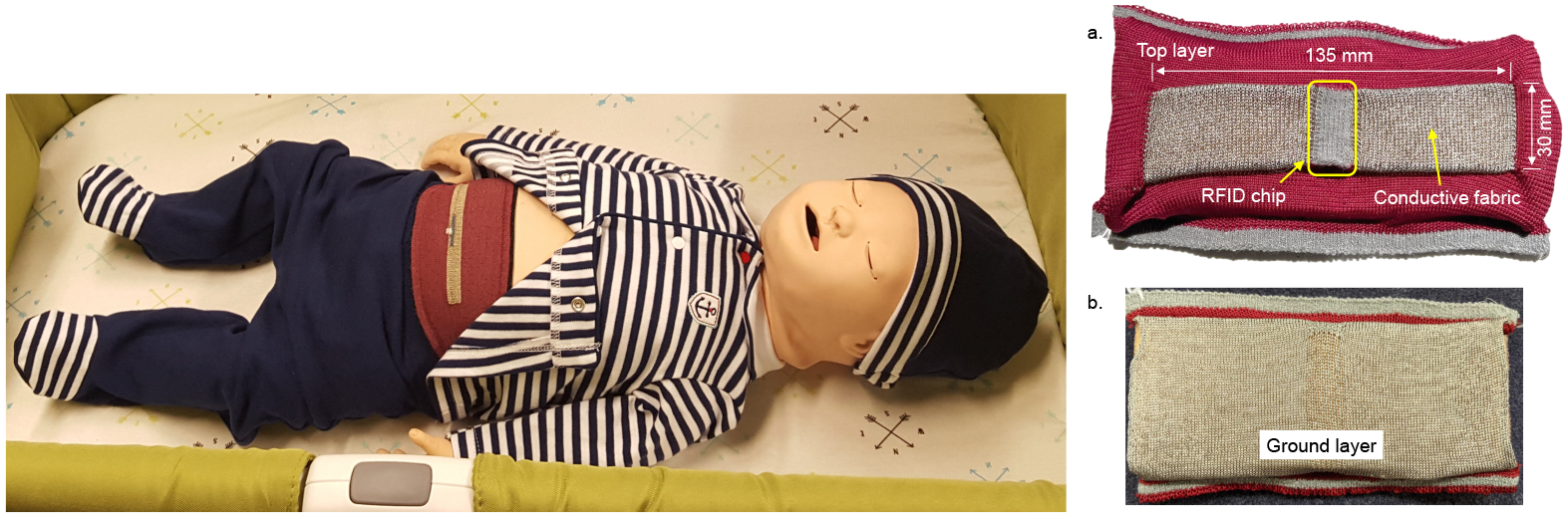
(**left**) A smart-fabric Bellyband and (**right**) Bellypatch can be integrated into wearable garments to enable wireless and passive biomedical monitoring in infants. (**a**) top and (**b**) bottom view of the Bellypatch.

We have studied the use of wearable technologies in the respiratory monitoring of infants. To this end, we use the Bellypatch (see Figure 1), a wearable smart garment that utilizes a knitted fabric antenna and passively reflects wireless signals without requiring a battery or wired connection [5–7]. The Bellypatch fabric stretches and moves as the infant breathes, contracts muscles, and moves about in space; the physical properties of the radio frequency (RF) energy reflected by the antenna change with these movements. These perturbations in RF-reflected properties enable detection and estimation of the infant’s respiration rate [8]. There are other possibilities of collecting heart rate [9], movement of the extremities [10], and detection of diaper moisture [11] using RF for medical practice.

The Bellypatch operates in the 900 MHz Industrial, Scientific, and Medical (ISM) frequency band using Radio Frequency Identification (RFID) interrogation. An RFID interrogator emits an interrogation signal multiple times per second (often 30–100 interrogations per second). In typical RFID technology deployments, only a single interrogation response is needed for inventory purposes; however, many interrogations are sent to overcome collisions among the responding RFID tags and other signal interference or loss. We exploit this redundant interrogation to sample the state of the antenna each time it is successfully interrogated. Specifically, we observe changes in the received signal strength indicator (RSSI), phase angle, and successful interrogation rate to estimate respiratory properties of the wearer’s state as well as other biosignals. The use of passive RFID enables a wireless and unobtrusive wearable device that requires no batteries to operate; the interrogation signal itself is sufficient to power the worn RFID tag for each interrogation. However, path loss, multipath fading, and collision mitigation among the tags in the field require signal denoising and interpolation of the received signal as well as intelligent algorithms for signal processing and estimation. Because these biomedical estimates require real-time or near-real-time processing and may take place on low-power embedded or portable systems, it is desirable to utilize techniques that place minimal constraints on power consumption, online training, and processing latency.

To this end, we propose a deep-learning-enabled respiratory classification approach. At the core of this approach is a five stage pipeline involving data collection and labeling, feature selection, deep learning model selection with hyperparameter optimization, model training and validation, and model testing and deployment. We use a Laerdal SimBaby programmable infant mannequin (see Figure 1) to collect respiratory data using the sensors attached on the Bellypatch. We use a 1-D convolutional neural network (1DCNN) for classification of the features extracted from the SimBaby sensor data. The 1DCNN model consists of one convolution layer, one polling layer, and three fully connected layers, achieving 97.15% classification accuracy (see Section 6.1). This is higher than state-of-the-art accuracy obtained using machine learning techniques such as Support Vector Machine (SVM), Logistic Regression (LR), and Random Forest (RF), all of which are proposed for the respiratory classification of infants. Hyperparameters of this 1DCNN model are selected using a Grid Search method, which is discussed in Section 3.4, and the model parameters are trained using the Backpropagation Algorithm [12]. Our model is trained using the respiratory data on an external work station. We also test the model in the workstation. Since we intend to deploy the trained network on a wearable device, we conduct energy efficiency explorations to find which approach provides the least power consumption and maximum accuracy. We show that the energy consumption of our baseline 1DCNN model is considerably higher, which makes it difficult to implement the same on the Bellypatch due to its limited power availability. Our goal is to minimize the energy consumption, thus allowing more processing within a given power budget. Therefore, we propose several quantization approaches involving limiting the bit-precision of the model parameters. We show that in order to achieve a significant reduction in energy, the model accuracy can be considerably lower. Therefore, model quantization may not be the best solution to implement respiratory classification on the Bellypatch.

Finally, we propose a novel respiratory classification solution enabled by spiking neural network (SNN) [13], which can be implemented on event-driven neuromorphic hardware such as TrueNorth [14], Loihi [15], and DYNAPs [16]. We perform design-space exploration using SNN parameters, obtaining SNN solutions with different accuracy and energy. We select a solution that leads to 93.33% accuracy with 18× lower energy than the baseline 1DCNN model. This SNN-based solution has similar accuracy as the best performing quantized CNN model with 4× lower energy. This is particularly useful for wearable devices that are used for bio signals monitoring using less energy such as the Human++ [17].

Overall, the SNN-based approach introduces two additional stages in our design pipeline: model conversion and SNN parameter tuning, making the overall approach a seven-stage pipeline. Using this seven stage design pipeline, we show that the accuracy is significantly higher than all prior solutions, with considerably lower energy, making this solution extremely relevant for the battery-less Bellypatch.

The remainder of this paper is organized as follows. Related works on respiratory classification are discussed in Section 2. The five-stage design pipeline is described in Section 3. Model quantization techniques are introduced in Section 4. The SNN approach to respiratory classification is formulated in Section 5. The proposed approach is evaluated in Section 6 and the paper is concluded in Section 7.

## 2. Related Work

Recently, machine learning-based respiratory classification techniques have shown significant promise as enablers for continuous respiratory monitoring of newborn infants. To this end, a Support Vector Machine (SVM)-based classifier is proposed in [18], achieving 82% classification accuracy. A Logistic Regression-based classifier is proposed in [19], achieving classification accuracy of 87.4%. An Ensemble Learning with Kalman filtering is proposed in [19] achieving 91.8% classification accuracy. All these techniques are proposed for respiratory classification using pulseoximeter data collected from infants, making these approaches the relevant state-of-the-art for our work. In Section 6, we compare our approach to these state-of-the-art approaches and show that the proposed approach is considerably better in terms of both classification accuracy and energy. Thermal imaging has also been proposed recently for the respiratory classification of infants [20]. The authors reported a precision and recall score of 0.92. We achieved a score of 0.98. Respiratory classification using acoustic sensors is proposed in [21]. An accuracy of 95.7% is reported. We achieved an accuracy of 97.15%.

Beyond respiratory classification, deep-learning-enabled techniques have been used extensively for health informatics [22]. For instance, sleep apnea classification is proposed using deep convolutional neural networks (CNNs) and long short-term memory (LSTM) in [23], achieving an accuracy of 77.2%. A deep learning approach using InceptionV3 CNN model is proposed in [24] to detect Alzheimer’s disease using brain images. The authors reported an area-under-curve (AUC) score of 0.98. CNN models are used in [25] to detect metastatic breast cancer in women. The authors reported an AUC score of 0.994. A CNN-based Arrhythmia classification is also proposed in [26], where the authors reported significant improvement in classification accuracy over state-of-the-art.

Finally, many recent SNN-based techniques have shown comparable and in some cases higher accuracy than their deep learning counterparts with significantly lower energy. An unsupervised SNN-based heartrate estimation is proposed in [27]. The authors reported a 1.2% mean average percent error (MAPE) with 35× reduction in energy. A spiking CNN architecture is proposed in [28] to classify heart-beats in human. The authors reported 90% reduction in energy with only 1% lower accuracy than a conventional CNN. SNN-based epileptic seizure detection is proposed in [29], where the authors reported an accuracy of 97.6% with a considerable reduction in energy.

To the best of our knowledge, this is the first work that uses SNN for respiratory classification in infants and shows that SNNs can achieve high accuracy (93.33% in our evaluation) with a considerable reduction in energy (18× lower energy compared to a baseline 1DCNN model).

## 3. Design Pipeline

**Figure 2.**
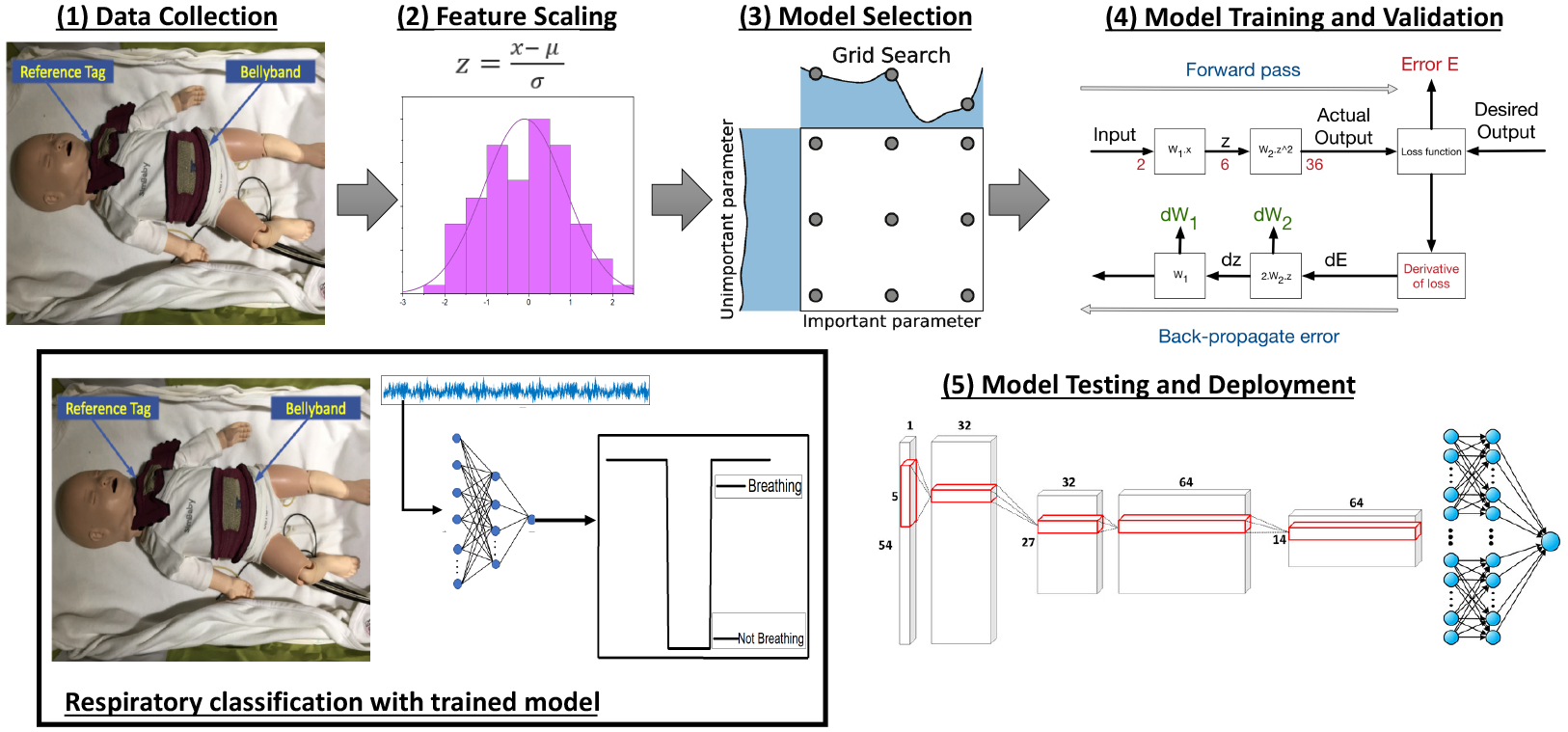
Design pipeline for respiratory classification using deep learning.

Figure 2 shows a high-level overview of the proposed respiratory classification approach using deep learning techniques. The design pipeline comprises five stages—(1) data collection, (2) feature selection, (3) deep learning model selection, (4) model training and validation, and (5) model testing and deployment. These stages are clearly indicated in the figure. Once a trained model is obtained using this approach, the model is used for the respiratory classification of streaming pulseoximeter data collected from the sensors on the Bellypatch. This is shown at the bottom-left corner of Figure 2.

In the following section, we describe the design pipeline stages.

### 3.1. Data Collection and Labeling

#### 3.1.1. Data Collection and Feature Selection

In this work, we use a Laerdal SimBaby programmable infant mannequin. The mannequin was programmed to breathe at a rate of 31 breaths per minute for variable time intervals, then to stop breathing for 30 s, 45 s, and 60 s, alternating between these states for a period of one hour. An RFID investigator (Impinj Speedway R420) was used to poll the Bellypatch wearable RFID tag and the antenna with a 900 MHz band RFID signal coming from the SimBaby. The RFID interrogator was also used to measure properties of the backscattered signal reflected from the RFID tag. The interrogator was positioned 1 foot from the mannequin, oriented above, astride, and at the feet. Interrogations were performed with a frequency of 90 Hz. RFID properties considered for model features include the Received Signal Strength Indicator (RSSI), interrogation frequency, and timestamp.

Each of these properties is affected in-band by the frequency of the original signal emitted by the interrogator. Under United States Federal Communications Commission (FCC) regulations, RFID interrogations must iterate (or channel hop) over 50 frequency channels in the 900 MHz band. In addition to perturbing the raw measurement observations at the interrogator, channel hopping poses challenges in computing higher order features from changes in the observed phase, because these features depend on observing changes in successive values of the phase under the assumption that they were observed from the same interrogation frequency. As a result, the observed Doppler shift is used to identify fine movements of the RFID tag, either in space or because of a strain force applied to the surrounding knit antenna.

The received signal strength from an interrogation is influenced by several factors as defined by the Radar Cross Section (RCS) formula in Equation (1) [30]. Specifically, the RCS relates changes in received signal power (*P*_*Rx*_) to the interrogation power (*P*_*Tx*_), the reader and tag gains (*G*_*reader*_ and *G*_*tag*_, respectively), the return loss (R), and the interrogation wavelength *λ* [19].


(1) $$P_{Rx}=P_{Tx}\cdot G_{\text{reader}}^{2}\cdot G_{tag}^{2}\cdot R\cdot {\left(\frac{\lambda }{4}\cdot \pi \cdot r\right)}^{4}$$

Some of these terms can be controlled by the interrogator configuration: for example, the interrogation transmitter power and antenna reader gain at the interrogator; however, the interrogation frequency changes due to channel hopping, and the receiving antenna gain (*G*_*tag*_) changes as the wearer stretches the antenna or moves about in space. Thus, the observed RSSI alone confounds several artifacts about the state of the transmission along with the state of the wearer. As a result, a higher order feature *ζ* is computed from the RSSI measure by accounting for the interrogation frequency.

We manipulate the RCS equation to arrange those terms related to wearer state on one side and set them as equal to those terms related to the interrogator configuration, as shown in Equation (2). Thus, we observe that the changes in the gain of the tag *G*_*tag*_ (resulting from movement or a strain force on the antenna), the distance r between the interrogator and the tag (resulting from movement), and return loss *R* (resulting from movement, strain force, fading, or multipath interference) are proportional to the interrogation wavelength lambda and observed *RSSI* measure *P*_*Rx*_, along with the interrogation power *P*_*Tx*_ and the reader gain *G*_*reader*_, which are held constant at the interrogator and interrogating antenna [5]. Specifically, *ζ* is defined as the ratio of the interrogation radius to the product of the antenna effective aperture and return loss, as shown in Equation (2), which represents the observed terms after fixing the transmit power, interrogator antenna gain, and interrogation frequency, given the observed RSSI of the reflected signal:

(2) $$\zeta =G_{tag}^{-2}\cdot r^{4}\cdot R^{-1}=P_{Tx}\cdot G_{\text{reader}}^{2}\cdot P_{Rx}^{-1}\cdot {\left(\frac{\lambda }{4\pi }\right)}^{4},$$

We remove a residual term $\delta =-10log_{10}\frac{f^{4}}{{\left(f-\left(0.5*10^{6}MHz\right)\right)}^{4}}\approx -0.00941$ to compensate for a sawtooth artifact resulting from quantization of the observed RSSI as the interrogation frequency changes among 50 discrete channels per FCC regulations in the United States.

In summary, we chose the following features for consideration during wireless respiratory state classification.

- Feature 1: Reflected signal strength as measured at the interrogator, *ζ* (*P*_*Rx*–*deoscillated*_)
- Feature 2: The difference between the current observed RSSI from the minimum RSSI value observed in the recent time window (*RSSI*_*from*_*min*)

We normalized RFID signal strength (*P*_*Rx*–*deoscillated*_) data by frequency to utilize the signal for respiratory analysis. The resulting time-series data were filtered and signal-processed to determine the mean power spectral density, derived from the amplitude of the oscillatory behavior observed in the signal during short time windows.

**Figure 3.**
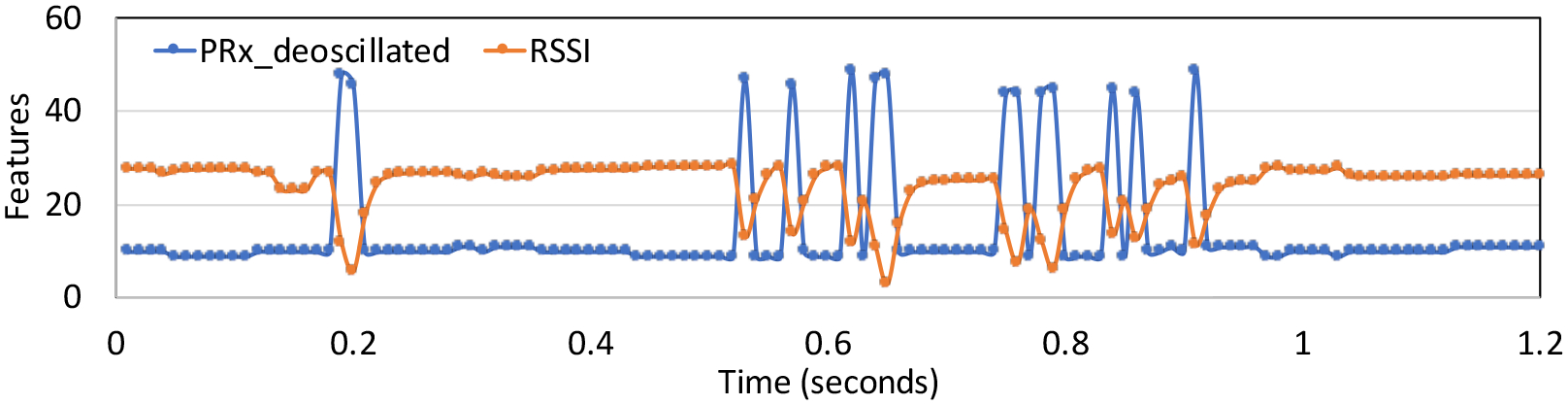
Variation of the two features PRx_deoscillated and RSSI_from_min for 1.2 s.

Figure 3 illustrates the two features (*P*_*Rx*–*deoscillated*_ and *RSSI*_*from*_*min*) over a time window of 1.2 s.

#### 3.1.2. Data Labeling

In case of supervised learning of human-activity recognition from sensor data, it is necessary to appropriately label the output. The dataset contained approximately 1685 samples per minute, obtained for 60 min, resulting in approximately one sample generated every 0.03 s. This indicated that the RFID interrogation frequency was approximately 28 Hz per RFID tag. For each sample, we have two features—*P*_*Rx*–*deoscillated*_ and *RSSI*_*from*_*min*.

**Figure 4.**
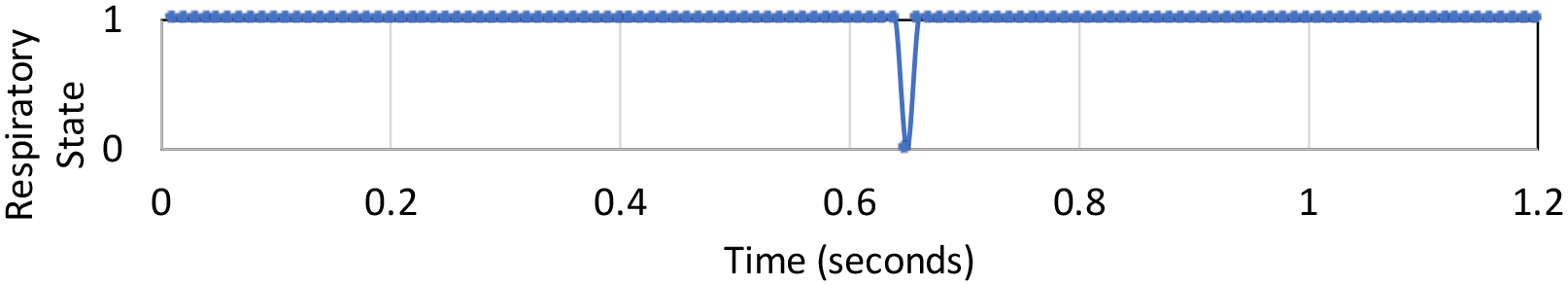
Respiratory state corresponding to features shown in Figure 3.

The observations were broken into time windows of 1 s with no overlap. Hence, each time window contained approximately 28 samples, with two sets of observations from the 2 features. We manually labeled the data collected from the two features as ‘1’ when the SimBaby is in breathing state and ‘0’ when it is in a non-breathing state. Over a period of one hour, we collect and label the dataset to form train and test samples representing binary respiratory state (1: Breathing, 0: Non-Breathing state). Figure 4 shows the respiratory state corresponding to the features of Figure 3 for 1.2 s.

Since there are only two features and two output classes, the problem we aim to solve is a bivariate time series binary classification one.

### 3.2. Feature Scaling

From the time series features extracted from each time window, we apply feature engineering to make the input vectors suitable for the classifier. For multivariate data, it is necessary to transform features with different scales to have uniform distribution, to ensure optimal performance of the classifiers. We first cleaned our feature set by filtering the missing values (NaN). The data with the features and the labels were loaded from two csv files and then we split the dataset 3:1 to form the training set and the testing set. After splitting the dataset, we scale the features before we fit it into our classifier, which is a one-dimensional convolutional neural network (1DCNN).

The two features in our dataset were scaled to a standard range and the distribution of the values was rescaled, so the mean was 0 and the standard deviation was 1. The method involved determining the distribution of each feature and subtracting the mean from each feature. Then we divide the values (after the mean has already been subtracted) of each feature by its standard deviation.

The standard score (*Z*) of a sample is given by Equation (3).

(3) $$Z=\frac{x-\mu }{\sigma },$$

where *x* is the sample value and *μ* and *σ* are the mean and standard deviation of all the samples, respectively. Feature standardization transforms the raw values into the standard scale that helps the model to extract salient signal information from the observations. After rescaling the variables, we reshape the data according to dimension expected by the convolution layer of the 1DCNN model.

### 3.3. Deep Learning Model Selection

A convolutional neural network (CNN) is a class of deep learning that uses a linear operation called convolution in at least one of its layers. Equation (4) represents a convolution operation, where *x* is the input and *w* represents the kernel, which stores parameters for the model. The output *s* is called the feature map of the convolution layer.


(4) s(t)=∫x(a)w(t−a)da

In a CNN, the first layer is a convolution layer that accepts a tensor as an input with dimensions based on the size of the data [31]. The second layer, or the first hidden layer, is formed by applying a kernel or filter that is a smaller matrix of weights over a receptive field, which is a small subspace of the inputs. Kernels apply an inner product on the receptive field, effectively compressing the size of the input space [12]. As the kernel strides across the input space, the first hidden layer is computed based on the weights of the filter. As a result, the first hidden layer is a feature map formed from the kernel applied on the input space. While the dimension of the kernel may be much smaller in size compared to the initial inputs of the convolution layer, the kernel must have the same depth of the input space. The inputs and convolution layers are often followed by rounds of activation, normalization, and pooling layers [12]. The precise number and combination of these layers are specific to the problem at hand.

**Figure 5.**
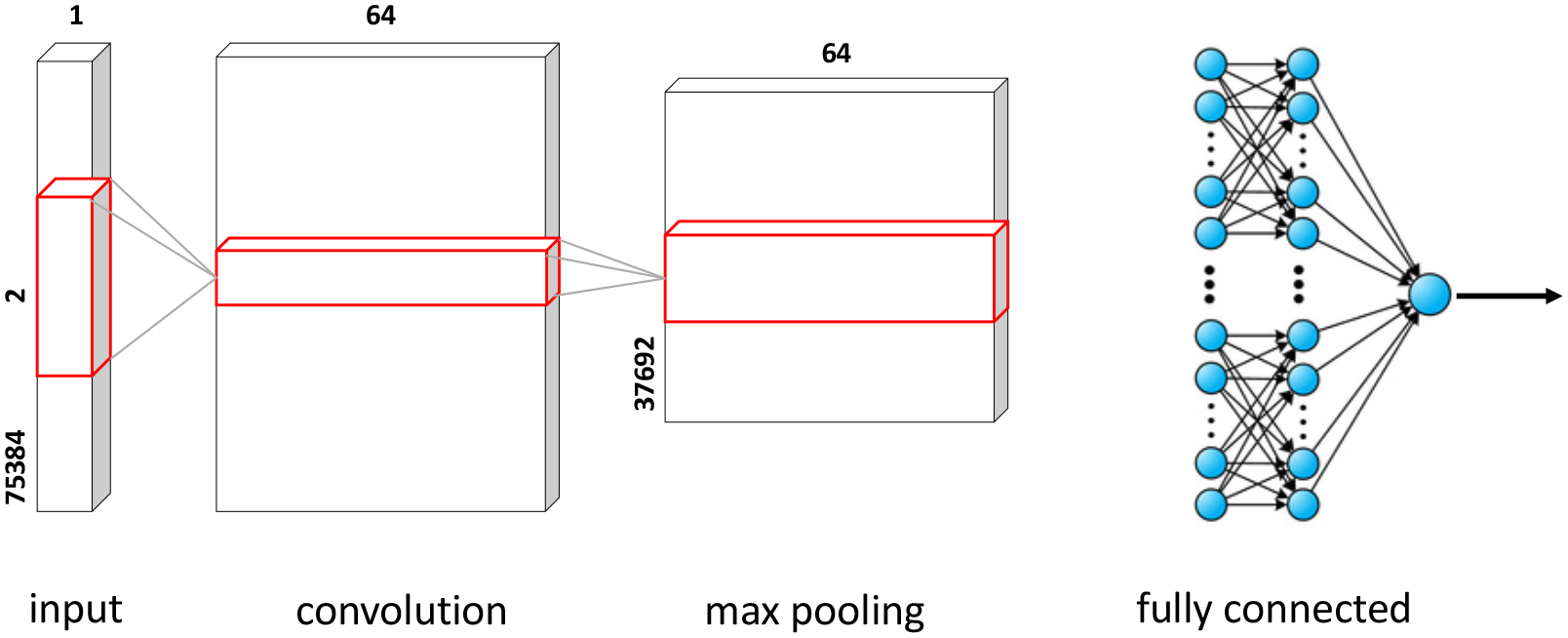
Our proposed 1DCNN architecture.

For the proposed respiratory classification problem, our CNN model consists of one convolution layer, which is activated by a rectified linear unit (ReLU). The ReLU activation is a suitable choice for non-linear transformation without the problem of vanishing gradient. The filter size is set to 64 and the kernel size to 1. This layer is followed by a one dimensional Max Pooling layer with a pool size and stride length of 1, each. The next layer is a Flattening layer followed by a Dropout layer. The Dropout layer randomly sets input neurons to 0 with a rate of 0.01 at each step during training time. This is done to prevent overfitting. The dropout layer is followed by two fully connected hidden layers. The first hidden layer consists of 200 neurons with ReLU activation, and the second hidden layer contains 100 neurons with a ReLU activation function and a Softmax function for the output layer. Softmax is a choice for the output layer, for output to be interpreted as normalized probabilities. Overall, the proposed CNN model uses one-dimensional convolutions and therefore, this model is referred to as *1DCNN*. Figure 5 shows the proposed 1DCNN architecture along with the dimension of each layer.

### 3.4. Hyperparameter Optimization

**Figure 6.**
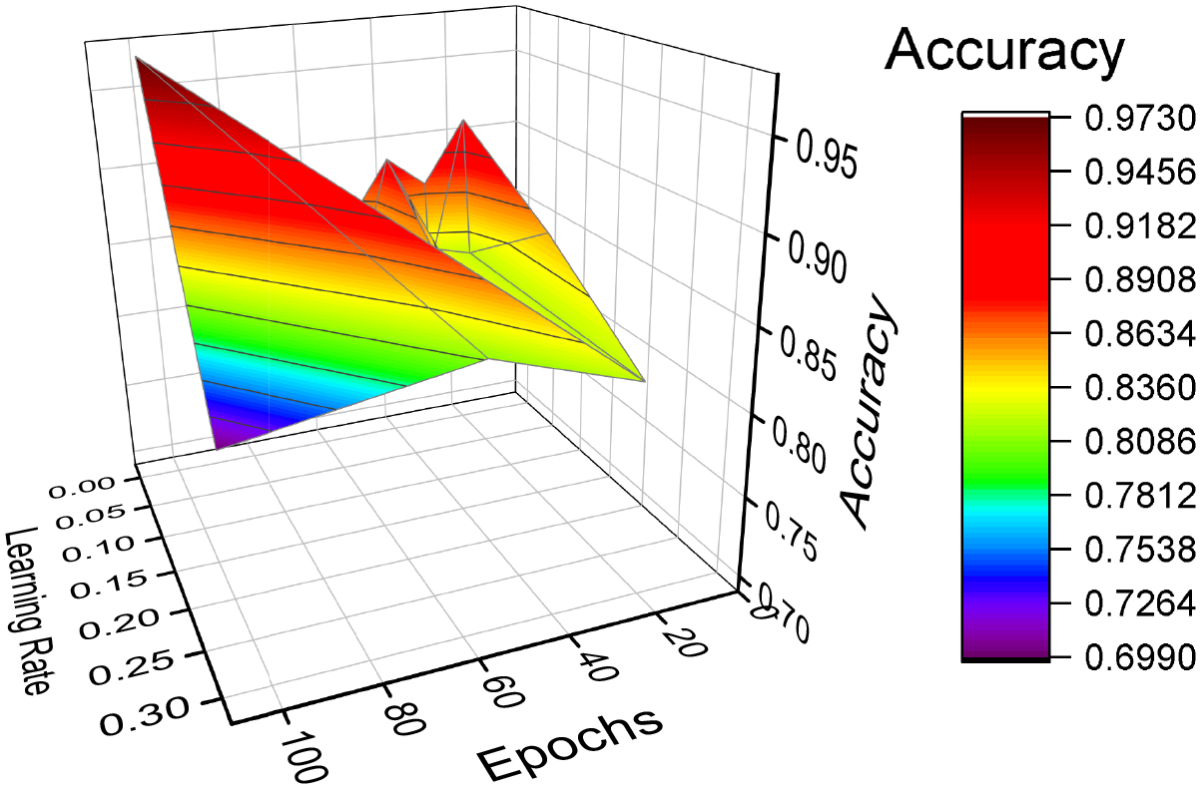
Hyperparameter selection.

In a CNN, the parameters in each layer whose values control the learning procedure are called hyperparameters. Grid search is a hyperparameter tuning technique that can build a model for every new combination of hyperparameters that is specified in the search space and evaluates each model for that combination. A machine learning algorithm *A*_*θ*_ can estimate a model *M*_*θ*_ ∈ *M* that minimizes a loss function L with its model regularization term R given by

(5) $$A_{\theta }\left(X_{\text{train}}\right)=\underset{M_{\theta }\in M}{\text{argmin}}L\left(M_{\theta },X_{\text{train}}\right)+R\left(M_{\theta },\theta \right),$$

where *X*_train_ is the training dataset, *M* is the set of all models, *θ* ∈ Θ is the chosen hyperparameter configuration, and Θ = {Θ_1_, Θ_2_, ⋯, Θ_*p*_} is the p-dimensional hyperparameter space of the algorithm. The optimal hyperparameter configuration θ* is calculated using the validation set as

(6) $$\theta^{*}=\underset{\theta \in \Theta }{\text{argmin}}L(A_{\theta }\left(X_{\text{train}},X_{\text{validation}}\right)=f_{D}(\theta ),$$

where *X*_validation_ is the validation set and *f*_*D*_ is the misclassification rate. The Grid search exhaustively searches through a grid of manually specified set of parameter values provided in a search space to find the accuracy obtained with each combination. We tuned the model based on the number of epochs (ranging from 10 to 100) and the learning rate for the Adam optimizer (0.001, 0.01, 0.1, 0.002, 0.02, 0.2, 0.003, 0.03, 0.3). We evaluated the accuracy as a performance metric for the different combinations. We also performed a Bayesian optimization that uses Bayes Theorem to tune the hyperparameters with a five-fold crossvalidation. We found the same parameters that are best fit to obtain best score. Figure 6 shows the selection of hyperparameters, where the accuracy is reported for different combinations of epochs and learning rates [32].

Table 1 summarizes the hyperparameters of the 1DCNN.

### 3.5. Model Training and Validation

We trained our 1DCNN model with 75,834 samples and used repeated k-fold cross-validation with 10 splits to validate our model performance. To improve the estimated performance, we repeated the cross-validation procedure multiple times and reported the mean result across all folds from all runs. This reported mean accuracy is expected to be a more accurate estimate of the true unknown underlying mean performance of the model on the dataset instead of a single run of k-fold cross-validation, ensuring less statistical noise. We also compute the standard error that provides an estimate of a given sample size of the amount of error that is expected from the sample mean to the underlying and unknown population mean. The standard error is calculated as

(7) $$\sigma_{\text{error}}=\frac{\sigma }{\sqrt{n}},$$

where *σ* is the sample’s standard deviation and *n* is the number of repeats. We obtained a validation classification accuracy of 87.78% with a standard error of 0.002 (see Section 6 for detailed evaluation). We defined Early Stopping as a regularization technique at the very beginning of declaring the model architecture. At end of every epoch, the training loop monitors whether the validation loss is no longer decreasing and once it is found no longer decreasing, the training is terminated. We enabled the patience parameter equal to 5 to terminate the training after epochs of no validation loss decrease. This is another measure to prevent the model from overfitting during training, alongside the addition of a Dropout layer. Without Early Stopping, the training would terminate only after reaching the maximum number of epochs.

### 3.6. Model Testing and Deployment

We deployed the trained model to test the performance on an unseen test set generated from the SimBaby to classify the respiratory states. The model was tested on 25,279 samples and achieved an accuracy of 97.15%, F1 Score of 0.98, AUC score of 0.98, sensitivity score of 0.96, and specificity score of 0.99. These performance metrics are defined in Section 6.1.

## 4. Model Quantization

CNN models consume a considerable amount of energy due to their high computational complexity. The high energy consumption is due to the large model size involving several thousand parameters. It is therefore challenging to deploy the inference, i.e., a trained CNN model on battery-powered mobile devices, such as smartphones and wearable gadgets due to their limited energy budget. To address this high energy overhead, energy-efficient solutions have been proposed to reduce the model size and computational complexity. Some common approaches include the pruning of network weights [33] and low bit precision networks [34]. We focus on the latter techniques. Specifically, we implement both bit precision weights and activations to reduce model sizes and computational power requirements. To perform the training of a CNN with low-precision weights and activations, we use the following quantization function to achieve a k-bit quantization [35].

(8) $$Z_{q}=Q\left(Z_{r}\right)=\frac{1}{2^{k}-1}\text{round}\left(\left(2^{k}-1\right)Z_{r}\right),$$

where *Z*_*r*_ ∈ [0, 1] is the full precision value and *Z*_*q*_ ∈ [0, 1] is the quantized value obtained using the k-bit quantization.

The quantization of weights is given by

(9) $$w_{q}=Q\left(\frac{\text{tanh}\left(w_{r}\right)}{2\cdot \text{max}\left(\left|\text{tanh}\left(w_{r}\right)\right|\right)}+\frac{1}{2}\right),$$

where *w*_*r*_ is the original weight using full precision and *w*_*q*_ is the quantized value using k-bit quatization.

The quantization of activations is given by

(10) $$x_{q}=Q(f(x)),$$

where *f*(*x*) = clip(*x*_*r*_, 0, 1) is the clip function bounding the activation function between 0 and 1.

In this paper, we apply both bit precision techniques for both weights and activations using quantization with the QKeras library, which is a quantization extension to the Keras [36]. It enables a drop-in replacement of layers that are responsible for creating parameters and activation layers like the Conv 1D, Dense layers. It facilitates arithmetic calculations by creating a deeply quantized version of a Keras model. We tag the variables, weights, and biases created by the Keras implementation of the model and the output of arithmetic layers by quantized functions. Quantized functions are specified as layer parameters and then passed as a cumulative quantization and activation function, QActivation. The quantized bits quantizer used above performs mantissa quantization using the following equation.

(11) $$\text{mantissa quantization}=2^{b-k+1}\cdot \text{clip}\left(\text{round}\left(x_{r}\cdot 2^{k-b-1}\right),-2^{k-1},2^{k-1}-1\right)$$

where *x* is the input given to the model, *k* is the number of bits for quantization, and *b* specifies how many bits of the bits are to the left of the decimal point.

We conduct our experiment to perform quantization of our Conv1D model using 2 bits, 4 bits, 8 bits, 16 bits, 32 bits, and 64 bits. We observe the performance accuracy increase with the increase in the quantization bits, with 2 bits achieving an 88.93% compared to using all the 64 bits achieving 97.15% (see the detailed results in Section 6.2).

The QTools functionality is used to estimate the model energy consumption for the different bit-wise quantization implementations. It estimates a layer-wise energy consumption for memory access and MAC operations in a quantized model derived from QKeras. This is helpful when comparing the power consumption of more than one model running on the same device. The model size is calculated as the number of model parameters multiplied by the number of bits used in each scenario. We observe that when we increase the number of bits, the model size increases as well as the accuracy, but so does the consumption of energy (pJ). This homogeneous replacement technique of Keras layers, with heterogeneous per-layer, per-parameter type precision, chosen from a wide range of quantizers, enabled quantization-aware training and energy-aware implementation to maximize the model performance given a situation of resource constraints, like detection of respiratory cessation on premature infants in critical care conditions, which is crucial for high-performance inference on wearables.

## 5. SNN-Based Respiratory Classification

**Figure 7.**
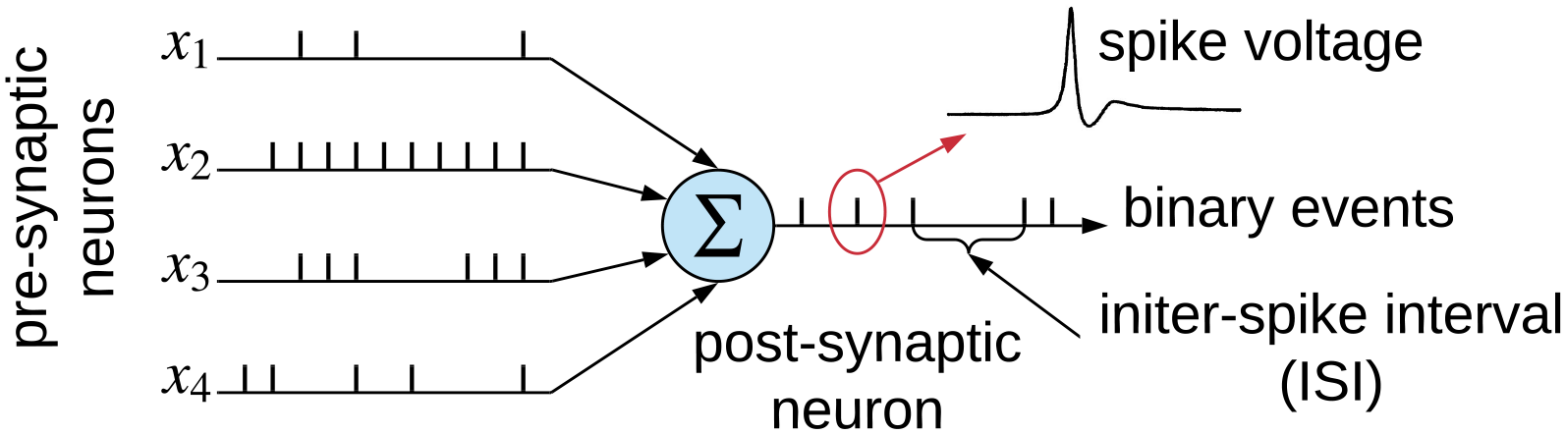
Integration of spike trains at the post-synaptic neuron from four pre-synaptic neurons in a spiking neural network (SNN). Each spike is a voltage waveform of ms time duration.

Spiking neural networks (SNNs), also known as the third generation of neural networks, are an interconnection of integrate-and-fire neurons that emulate the working principle of a mammalian brain [13]. SNNs enable powerful computations due to their spatio-temporal information-encoding capabilities. In an SNN, spikes (i.e., current) injected from pre-synaptic neurons raise the membrane voltage of a post-synaptic neuron. When the membrane voltage crosses a threshold, the post-synaptic neuron emits a spike that propagates to other neurons. Figure 7 shows the integration of spike train from four pre-synaptic neurons connected to a post-synaptic neuron via synapses.

SNNs can implement many machine learning approaches such as supervised, unsupervised, reinforcement, few-shot, and lifelong learning. Due to their event-driven activation, SNNs are particularly useful in energy-constrained platforms such as wearable and embedded systems. Recent works demonstrate a significant reduction in memory footprint and energy consumption in SNN-based heart-rate estimation [27], heartbeat classification [28,37], speech recognition [38], and image processing [39].

**Figure 8.**
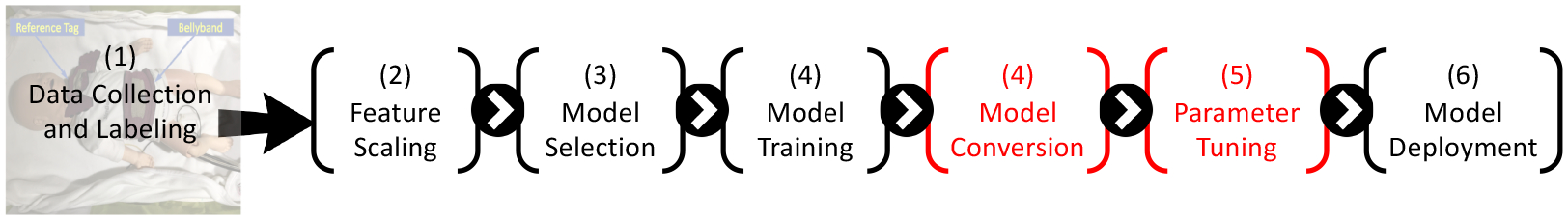
Seven-stage pipeline, including the two new stages to process and optimize the SNN model.

To integrate SNN-based respiratory classification into our design pipeline, we introduce two additional stages—model conversion and SNN parameter tuning—before the SNN model is deployed to perform classification from live data collected from the SimBaby. Figure 8 shows the new design pipeline.

### 5.1. Model Conversion

In this work, the 1DCNN architecture is converted to SNN in order to execute it on a neuromorphic hardware such as Loihi [15]. The conversion steps are briefly discussed below.

1. ReLU Activation Functions: This is implemented as the approximate firing rate of a leaky integrate and fire (LIF) neuron.
2. Bias: A bias is represented as a constant input current to a neuron, the value of which is proportional to the bias of the neuron in the corresponding analog model.
3. Weight Normalization: This is achieved by setting a factor *λ* to control the firing rate of spiking neurons.
4. Softmax: To implement softmax, an external Poisson spike generator is used to generate spikes proportional to the weighted sum accumulated at each neuron.
5. Max and Average Pooling: To implement max pooling, the neuron which fires first is considered to be the winning neuron, and therefore, its responses are forwarded to the next layer, suppressing the responses from other neurons in the pooling function. To implement average pooling, the average firing rate (obtained from total spike count) of the pooling neurons are forwarded to the next layer of the SNN.
6. 1-D Convolution: The 1-D convolution is implemented to extract patterns from inputs in a single spacial dimension. A 1xn filter, called a kernel, slides over the input while computing the element-wise dot-product between the input and the kernel at each step.
7. Residual Connections: Residual connections are implemented to convert the residual block used in CNN models such as ResNet. Typically, the residual connection connects the input of the residual block directly to the output neurons of the block, with a synaptic weight of ‘1’. This allows for the input to be directly propagated to the output of the residual block while skipping the operations performed within the block.
8. Flattening: The flatten operation converts the 2-D output of the final pooling operation into a 1-D array. This allows for the output of the pooling operation to be fed as individual features into the decision-making fully connected layers of the CNN model.
9. Concatenation: The concatenation operation, also known as a merging operation, is used as a channel-wise integration of the features extracted from two or more layers into a single output.

**Figure 9.**
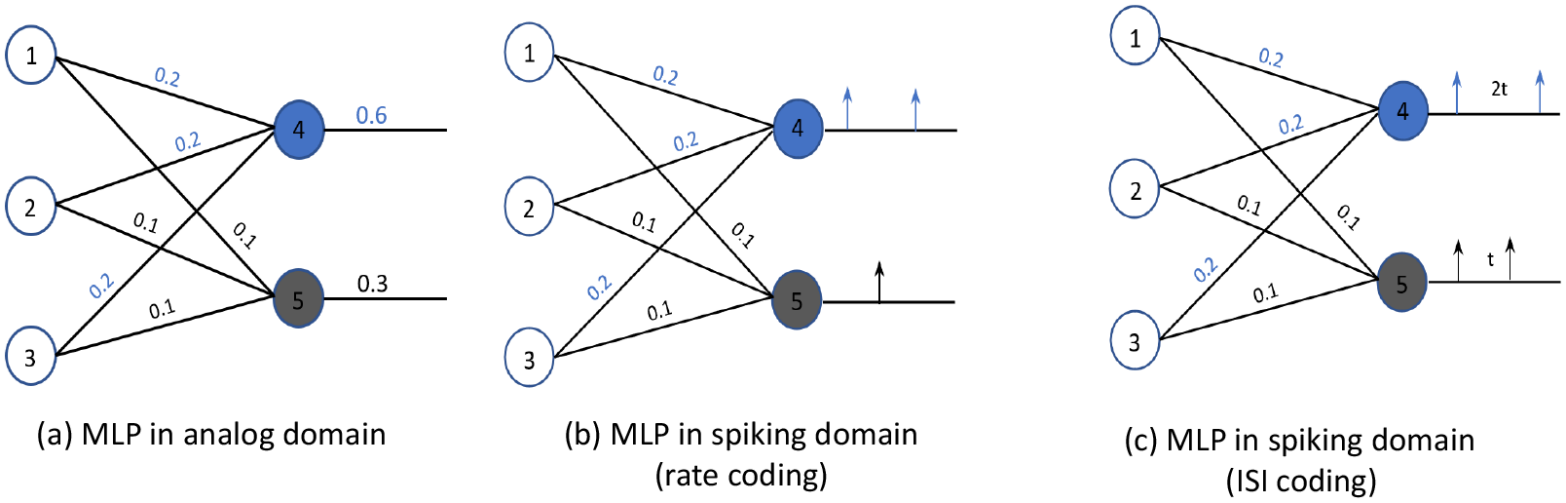
Example of converting an analog MLP to its spiking equivalent.

We now briefly elaborate how an analog operation such as Rectified Linear Unit (ReLU) is implemented using SNN. The output *Υ* of a ReLU activation function is given by

(12) $$Y=\text{max }0,\underset{i}{\sum }w_{i}*x_{i},$$

where *w*_*i*_ is the weight and *x*_*i*_ is the activation on the *i*^th^ synapse of the neuron. To map the ReLU activation function, we consider a particular type of spiking neuron model known as an integrate and fire (IF) neuron model. The IF spiking neuron’s transfer function can be represented as

(13) $$v_{m}(t+1)=v_{m}(t)+\underset{i}{\sum }w_{i}*x_{i}(t),$$

where *v*_*m*_(*t*) is the membrane potential of the IF neuron at time *t*, *w*_*i*_ is the weight, and *x*_*i*_(*t*) is the activation on the *i*^th^ synapse of the neuron at time *t*. The IF spiking neuron integrates incoming spikes (*X*_*i*_) and generates an output spike (*Υ*_spike_) when the membrane potential (*v*_*m*_) exceeds the threshold voltage (*v*_th_) of the IF neuron. Therefore, by ensuring that the output spiking rate *Υ*_spike_ is proportional to the ReLU activation *Υ*, i.e., *Υ*_spike_ ∝ *Υ*, we accurately convert the ReLU activation to the spike-based model. To further illustrate this, we consider the multi-layer perceptron (MLP) of Figure 9a and its SNN conversion using rate-based encoding (Figure 9b) and inter-spike interval (ISI) encoding (Figure 9c).

In Figure 9a, neurons 1, 2, and 3 are the input neurons and neurons 4 and 5 are the output neurons. To keep the model simple, let us consider the case where the activations of the input neurons 1, 2, and 3 are equal to 1. Using Equation (12), we know that the output of neurons 4 and 5 are 0.6 and 0.3, respectively. Figure 9b,c shows the mapped SNN model, using rate-based and inter-spike interval encoding schemes, respectively. In the rate-based model in Figure 9b, the rate of spikes generated is expected to be proportional to the output of neurons 4 and 5 in the MLP. In the case of the ISI-based SNN model, the inter-spike interval of the spikes generated by neurons 4 and 5 is expected to be proportional to the output generated in the MLP, as shown in Figure 9c.

### 5.2. SNN Mapping to Neuromorphic Hardware

The SNN model generated using the conversion approach is analyzed in CARLsim [40] to generate the following information.

- Spike Data: the exact spike times of all neurons in the SNN model.
- Weight Data: the synaptic strength of all synapses in the SNN model.

**Figure 10.**
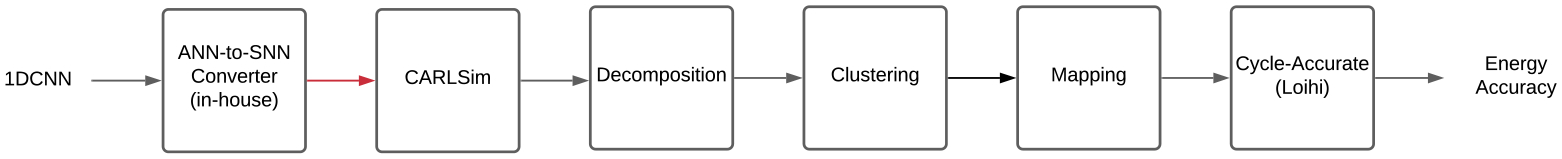
The NeuroXplorer framework [41].

The spike and weight data of a trained SNN form the SNN workload, which is used in the NeuroXplorer framework [41] to estimate the energy consumption. Figure 10 shows the NeuroXplorer framework.

The framework inputs the 1DCNN model and estimates the accuracy and energy consumption of the model on a neuromorphic hardware. Internally, NeuroXplorer first converts the 1DCNN to SNN using the steps outlined before. It then simulates the SNN using CARLsim. The extracted workload is first decomposed using the decomposition approach presented in [42]. This is to ensure that the workload can fit on to the resource-constraint hardware.

Typically, neuromorphic hardware is designed with tile-based architecture [43], where each tile can accommodate only a limited number of neurons and synapses. The tiles are interconnected using a shared interconnect such as Network-On-Chip (NoC) [44] or Segmented Bus [45]. Therefore, to map an SNN into a tile-based neuromorphic hardware, the model is first partitioned into clusters, where each cluster consists of a proportion of the neurons and synapses of the original machine learning model [46]. Each cluster can then fit onto a tile of the hardware. Then, the clusters are mapped to the tiles to optimize one or more hardware metrics such as energy [47,48], latency [49–53], circuit aging [54–59], and endurance [60–62]. We use the energy-aware mapping technique of [48].

Once the clusters of the converted 1DCNN model are placed with the resources of the neuromorphic hardware, we perform cycle-accurate simulations using NeuroXplorer, configured to simulate the Loihi neuromorphic system. Table 2 shows the hardware parameters that are configured in NeuroXplorer.

### 5.3. SNN Parameter Tuning

Unlike the baseline 1DCNN architecture, where model hyperparameters are explored only during model training, SNNs allow parameter tuning on the trained (and converted) model, such that the energy and accuracy space could be explored to generate a solution that satisfies the given energy and accuracy constraints of the target wearable platform. To explore such exploration capabilities, we analyze the dynamics of SNNs.

The membrane potential of a neuron at time *t* can be expressed as [13]

(14) $$u(t)=u_{0}+a\int_{0}^{t}D(s)\cdot w\cdot \sigma (t-s)ds,$$

where *u*_0_ is the initial membrane potential, *a* is a positive constant, *D*(*s*) is a linear filter, *w* is the synaptic weight and *σ* represents a sequence of *N* input spikes, which can be expressed using the Dirac delta function as

(15) $$\sigma (t)=\overset{N}{\underset{i=1}{\sum }}\delta \left(t-t_{i}\right)$$

The membrane potential of a neuron increases upon the arrival of an input spike. Subsequently, the membrane potential starts to decay during the inter-spike interval (ISI). When the neuron is subjected to an input spike train, the membrane voltage keeps rising, building on the undissipated component. When the membrane potential crosses a threshold (*V*_*th*_), the neuron emits a spike, which then propagates to other neurons of the SNN. The spike rate of a neuron can be controlled using this threshold. If the threshold is set too high, fewer spikes will be generated, meaning that not only will the energy be lower but also the accuracy, because spikes encode information in SNNs. Therefore, by adjusting the threshold, the design space of accuracy and energy can be explored (see Section 6.3).

## 6. Results and Discussions

All simulations are performed on a workstation, which has AMD Threadripper 3960X with 24 cores, 128 MB cache, 128 GB RAM, and 2 RTX3090 GPUs. Keras [36] is used to implement the baseline 1DCNN, which uses TensorFlow backend [64]. QKeras [65] is used for training and testing the quantized neural network. Finally, CARLsim [40] is used for SNN function simulations.

We present our respiratory classification results organized into (1) results for the baseline 1DCNN model (Section 6.1), (2) results using quantization (Section 6.2), and (3) SNN-specific results (Section 6.3).

### 6.1. Baseline 1DCNN Performance

In this section, we evaluate the performance of the proposed 1DCNN specified using the following metrics.

- Top-1 Accuracy: This is the conventional accuracy and it measures the proportion of test examples for which the predicted label (i.e., respiratory state) matches the expected label. To formulate top-1 accuracy, we introduce the following definitions.
  - True Positives (TP): For binary classification problems, i.e., ones with a yes/no outcome (such as the case of respiratory classification), this is the total number of test examples for which the value of the actual class is yes and the value of predicted class is also yes.
  - True Negatives (TN): This is the total number of test examples for which the value of the actual class is no and the value of the predicted class is also no.
  - False Positives (FP): This is the total number of test examples for which the value of the actual class is no but the value of the predicted class is yes.
  - False Negatives (FN): This is the total number of test examples for which the value of the actual class is yes but the value of the predicted class is no.
(16) $$\text{Top-}1\text{ Accuracy}=\frac{TP+TN}{TP+FP+FN+TN}$$
- F1 Score: To formulate the F1 score, we introduce the following definitions.
  - Precision: This is the ratio of correctly predicted positive observations to the total predicted positive observations, i.e.,
(17) $$\text{Precision}=\frac{TP}{TP+FP}$$
  - Recall: This is the ratio of correctly predicted positive observations to the all observations in actual class, i.e.,
(18) $$\text{Recall}=\frac{TP}{TP+FN}$$
  The F1 score conveys the balance between the precision and the recall. It is calculated as the weighted average of precision and recall, i.e.,
(19) $$\text{F}1\text{ Score}=\frac{2*(\text{Recall}*\text{Precision})}{\left(\text{Recall}+\text{Precision}\right)}$$
- AUC: In machine learning, a receiver operating characteristic (ROC) curve is a graphical plot that illustrates the diagnostic ability of a binary classifier as its discrimination threshold is varied. The area under curve (AUC) measures the two-dimensional area underneath the ROC curve. AUC tells how much the model is capable of distinguishing between classes. The higher the AUC, the better the model is at predicting yes classes as yes and no classes as no.
- Sensitivity: This is the true positive rate, i.e., how often the model correctly generates a yes out of all the examples for which the value of actual class is yes. Sensitivity is formulated as
(20) $$\text{Sensitivity}=\frac{TP}{TP+FN}$$
- Specificity: This is the true negative rate, i.e., how often the model correctly generates a no out of all the examples for which the value of actual class is no. Specificity is formulated as
(21) $$\text{Specificity}=\frac{TN}{TN+FP}$$

Table 3 compares the classification performance using the proposed 1DCNN against three state-of-the-art approaches: (1) Support Vector Machine (SVM) classifier of [18], (2) Logistic Regression (LR) classifier of [19], and (3) Random Forest classifier of [19]. We make the following four key observations.

First, the proposed 1DCNN has the highest top-1 accuracy of all the evaluated techniques (higher top-1 accuracy is better). The top-1 accuracy of 1DCNN is better than SVM by 5.2%, LR by 6.0%, and RF by 4.0%.

Second, the proposed 1DCNN has the highest F1 score of all the evaluated techniques (higher F1 score is better). The F1 score of 1DCNN is higher than SVM by 7.7%, LR by 7.7%, and RF by 6.5%.

Third, the proposed 1DCNN has the highest AUC of all the evaluated techniques (higher AUC score is better). The AUC score of 1DCNN is higher than SVM by 6.2%, LR by 8.5%, and RF by 8.5%.

Fourth, the proposed 1DCNN has the highest sensitivity of all the evaluated techniques (higher sensitivity score is better). The sensitivity score of 1DCNN is higher than SVM by 3.2%, LR by 6.7%, and RF by 4.3%.

Finally, the proposed 1DCNN has the highest specificity of all the evaluated techniques (higher specificity score is better). The specificity score of 1DCNN is higher than SVM by 7.6%, LR by 7.6%, and RF by 6.4%.

The reason for high performance using the proposed 1DCNN model is two-fold. First, we perform intelligent feature selection from the data collected using sensors on the SimBaby programmable infant mannequin. Second, we perform hyperparameter optimization with neural architecture search to generate a model that gives the highest classification accuracy using the selected hyperparameters.

**Figure 11.**
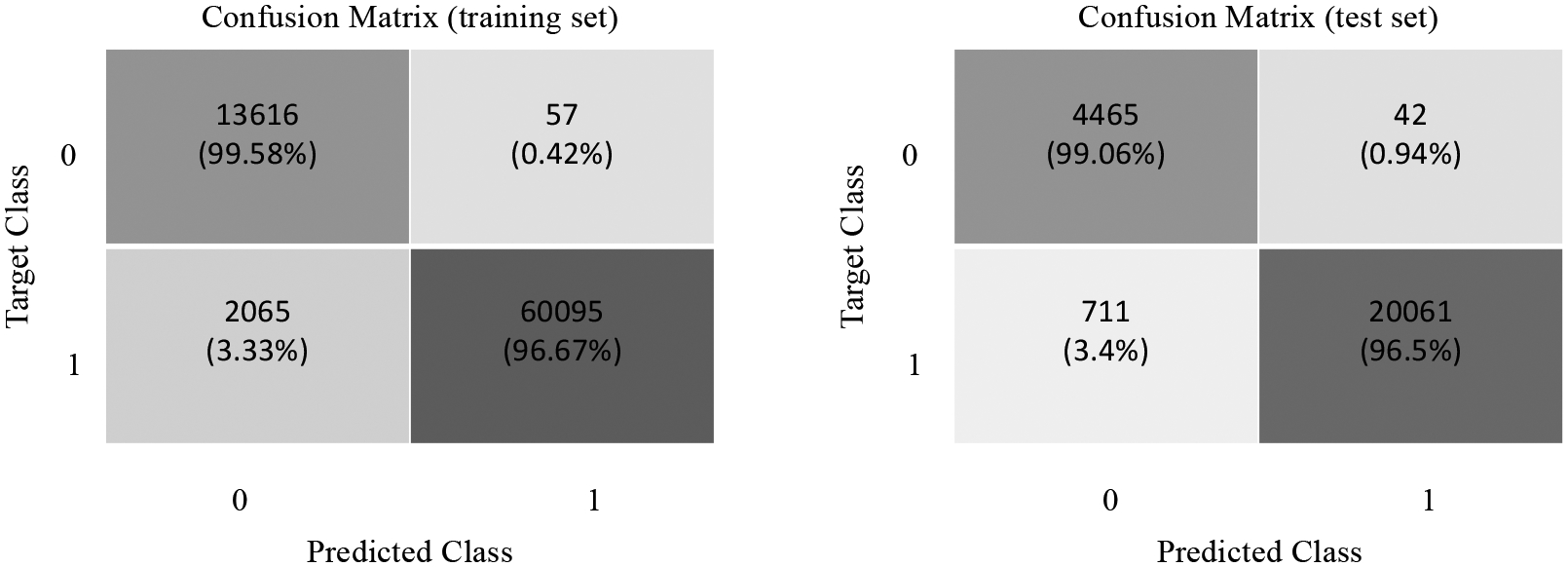
Confusion matrix for the 1DCNN model.

To give further insight to the improvement, Figure 11 shows the confusion matrix obtained for the training and test sets. We observe that the proposed 1DCNN model has very low false positives and false negatives, which are critical for respiratory classification in premature newborn infants.

### 6.2. Quantization Results

**Figure 12.**
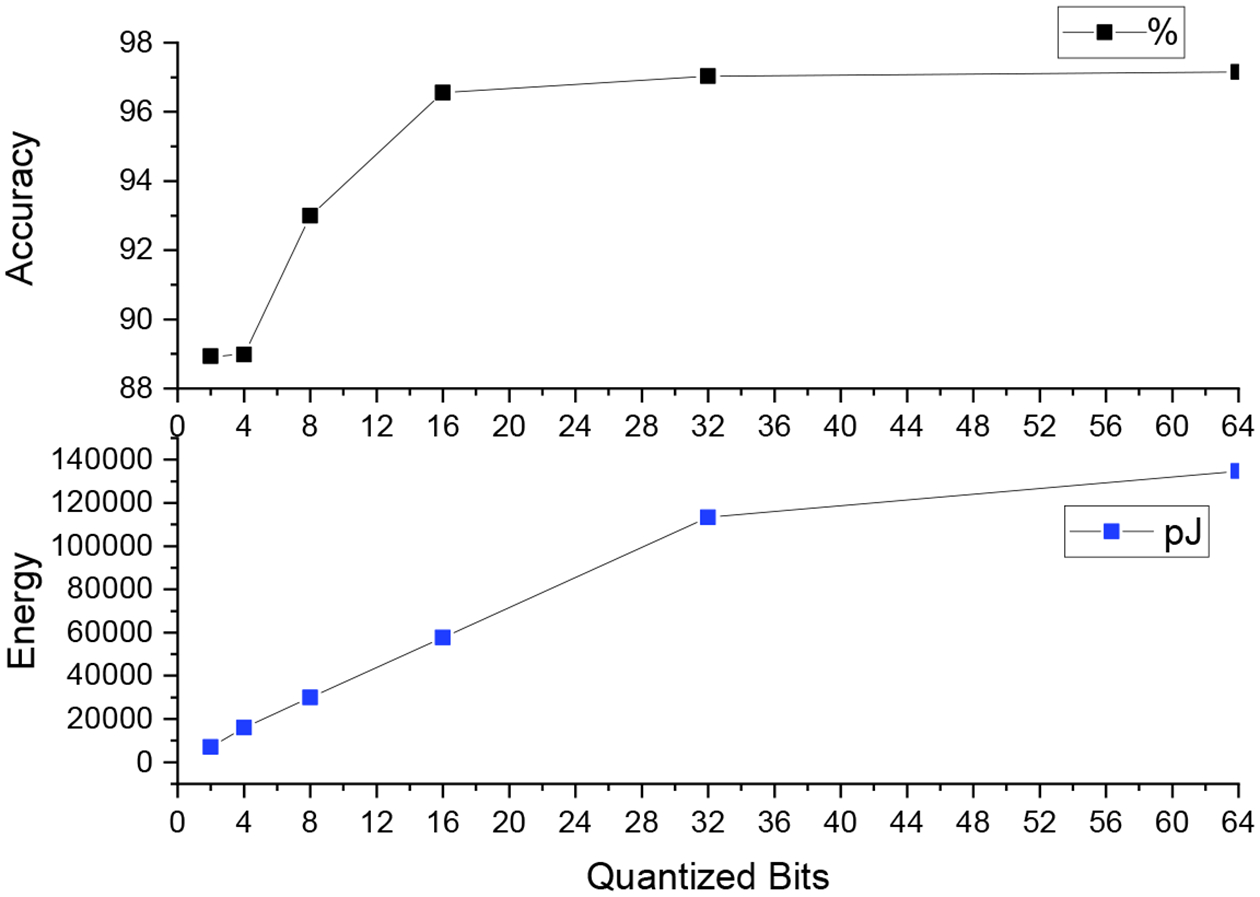
Relationship between quantization bits, accuracy, and energy consumption.

Table 4 and Figure 12 reports the top-1 accuracy (%), energy (in pJ), and model size (in bits) with 2-bit, 4-bit, 8-bit, 16-bit, and 32-bit precision for the model parameters. For comparison, we have included results using the baseline 1DCNN, which uses full 64-bit precision for the model parameters. We make the following three key observations.

First, the top-1 accuracy reduces with a reduction in the bit precision (higher accuracy is better for respiratory classification in premature newborn infants). With 2-bit, 4-bit, 8-bit, 16-bit, and 32-bit precision, the top-1 accuracy is lower than the baseline 64-bit precision by 8.5%, 8.4%, 4.3%, 0.6%, and 0.1%, respectively. The top-1 accuracy with 32-bit precision is comparable to 64-bit precision.

Second, energy reduces with a reduction of the bit precision (lower energy is better for respiratory classification in wearables due to limited battery, as we mentioned in Section 1). With 2-bit, 4-bit, 8-bit, 16-bit, and 32-bit precision, energy is lower than the baseline 64-bit precision by 94.7%, 88.1%, 77.8%, 57.2%, and 15.8%, respectively. These results show the significant reduction in energy achieved using quantization. Lower energy leads to longer battery life in wearables. Finally, model size also reduces with a reduction in the bit precision (lower model size is better for wearables due to their limited storage availability). With 2-bit, 4-bit, 8-bit, 16-bit, and 32-bit precision, energy is lower than the baseline 64-bit precision by 96.8%, 93.7%, 87.5%, 75.0%, and 50.0%, respectively.

We conclude that to reduce energy and model size for respiratory classification in wearables, quantization techniques can lead to a significant reduction in accuracy.

### 6.3. SNN-Related Results

Table 5 reports the top-1 accuracy and energy results using the proposed SNN-based approach compared to the baseline 1DCNN, 2-bit, and 8-bit quantized model. We make the following three key observations.

First, the top-1 accuracy of the proposed SNN is only 4% lower than the baseline 1DCNN model with 18× lower energy. Second, compared to the 2-bit quantized model, the top-1 accuracy is 5% higher, while the energy is only 2% higher. Finally, compared to the 8-bit quantized model, the top-1 accuracy is comparable while the energy is 4× lower. We conclude that SNN-based respiratory classification achieves the best tradeoff in terms of top-1 accuracy and energy. To achieve similar accuracy, SNN can lead to a 4× reduction in energy, which is a critical consideration for respiratory classification on wearables. The following results are reported to give further insight into these improvements.

#### 6.3.1. SNN Accuracy Compared to 1DCNN

**Figure 13.**
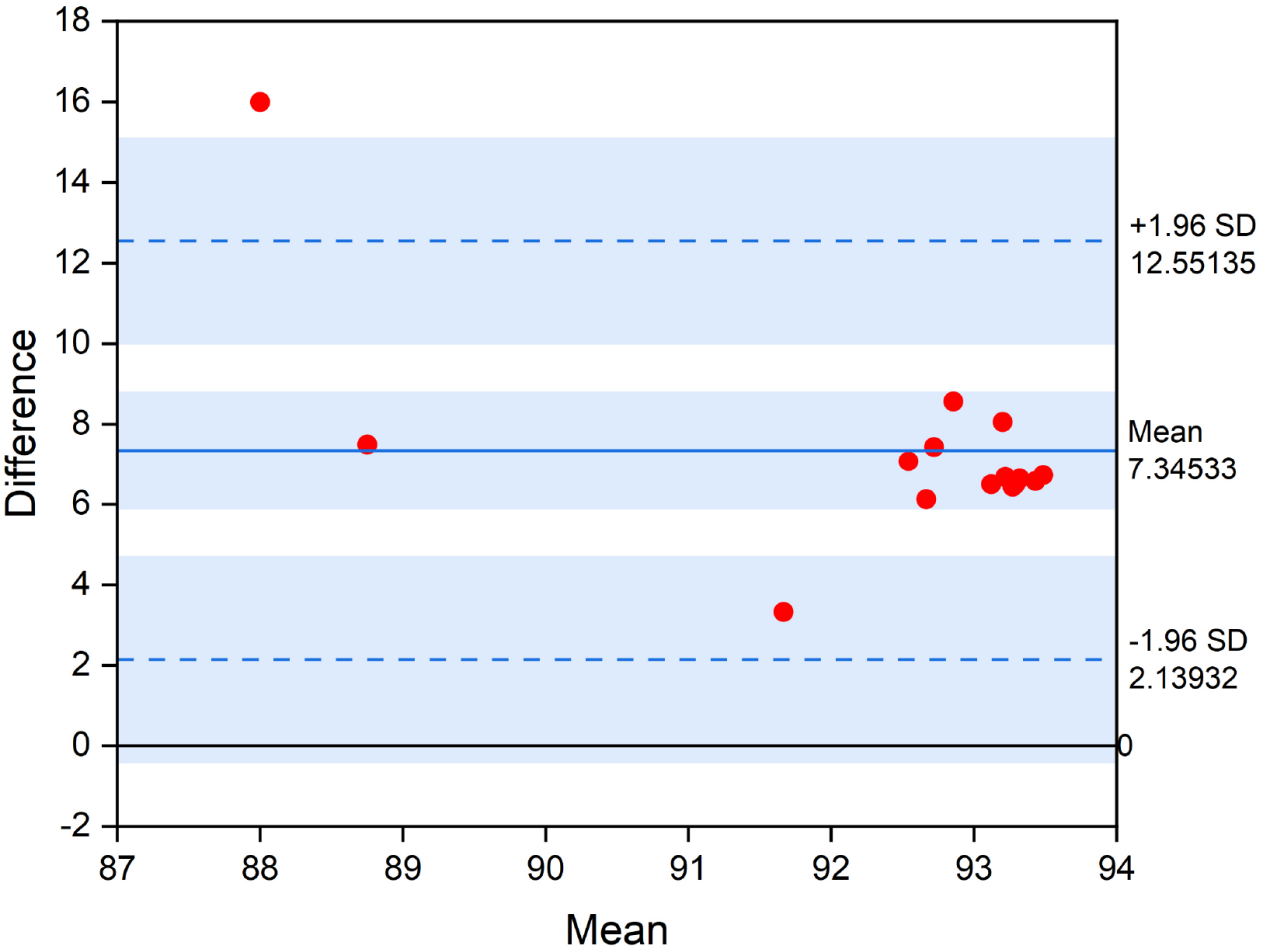
Bland–Altman plot comparing the accuracy of different SNN solutions against the baseline 1DCNN model.

Figure 13 shows the Bland–Altman plot comparing the accuracy of SNN solution against the baseline 1DCNN model. Bland–Altman plots are extensively used to evaluate the agreement among two models, each of which produced some error in their predictions. As can be seen from the plot, the average accuracy difference between the 1DCNN and the converted SNN is 7.3%, while the minimum and maximum accuracy difference are 2.1% and 12.5%, respectively.

#### 6.3.2. Design Space Exploration with SNN Parameters

We perform design-space explorations to identify SNN model parameters that give the best tradeoff in terms of energy and accuracy.

**Figure 14.**
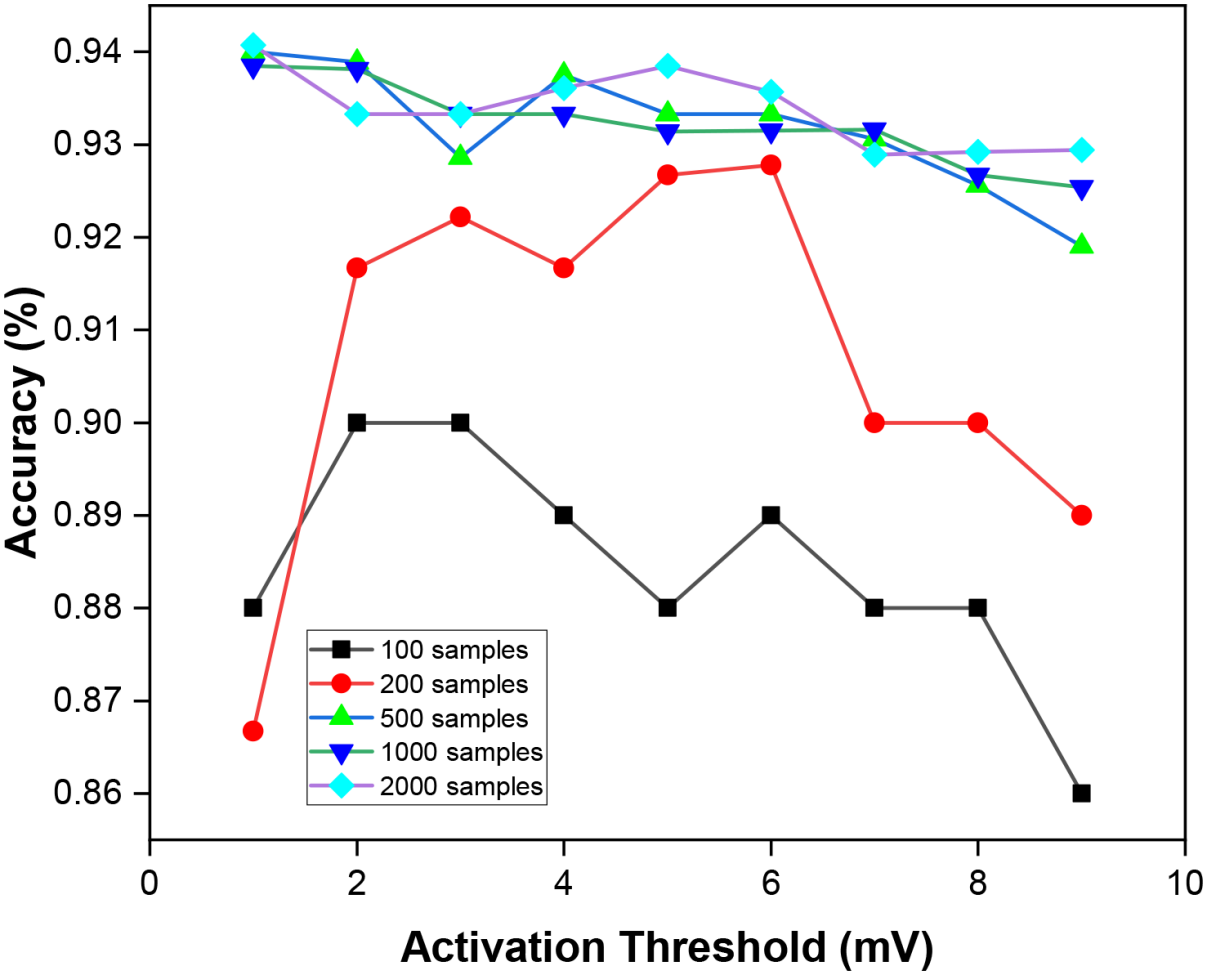
Accuracy impact with varying the activation.

In spiking neural networks, a spike is not fired by a neuron unless the specified activation threshold voltage is attained. This implies that the larger the firing threshold voltage, the more selectively a neuron is fired while communicating between each layer. To demonstrate this, Figure 14 shows the variation in accuracy as a function of the activation threshold (*V*_*th*_) (see Section 5.3).

In the figure, we vary the activation threshold across different number of test samples. We observe that for a smaller number of test samples, as we increase the threshold voltage for firing spikes, the performance accuracy varies highly. However, test samples with a larger size, the performance accuracy does not vary as much when we increase the threshold voltage.

For larger test sample sizes (500, 1000, 2000, etc.), the performance accuracy varies between 92% and 94%. When we use small sample sizes (e.g., 100 and 200), the prediction accuracy varies between 86% and 92%. We observe that the firing threshold is most ideal when set within 1–3 mV for smaller test samples and within 1–5 mV for larger test samples.

## 7. Conclusions

We propose a deep-learning-enabled wearable monitoring system for premature newborn infants, where respiratory cessation is predicted using signals that are collected wirelessly from a non-invasive wearable Bellypatch put on the infant’s body. To this end, we developed an end-to-end design pipeline involving five stages data collection, feature scaling, model selection, training, and deployment. The deep learning model is a 1D convolutional neural network (1DCNN), the parameters of which are tuned using a grid search methodology. Our design achieved 97.15% accuracy compared to state-of-the-art statistical approaches. To address the limited energy in wearable settings, we evaluate model compression techniques such as quantization. We show that such techniques can lead to a significant reduction in respiratory classification accuracy in order to minimize energy. To address this important problem, we propose a novel spiking neural network (SNN)-based respiratory classification technique, which can be implemented efficiently on an event-driven neuromorphic hardware. SNN-based respiratory classification involves two additional pipeline stages: model conversion and parameter tuning. Using respiration data collected from a Laerdal SimBaby programmable infant mannequin, we demonstrate 93.33% respiratory classification accuracy with 18× lower energy compared to the conventional 1DCNN model. We conclude that SNNs have the potential to implement respiratory classification and other machine learning tasks on energy-constrained environments such as wearable systems.

## Figures and Tables

**Table 1.** Summary of hyperparameters for the proposed 1DCNN model.

Learning rate	0.001
Batch size	5
Optimizer	Adam
Data shuffle	per epoch
Maximum epochs	100

**Table 2.** Major simulation parameters extracted from Loihi [15].

Neuron technology	16 nm CMOS (original design is at 14 nm FinFET)
Synapse technology	HfO_2_-based OxRRAM [63]
Supply voltage	1.0 V
Energy per spike	23.6 pJ at 30 Hz spike frequency
Energy per routing	3 pJ
Switch bandwidth	3.44 G. Events/s

**Table 3.** Comparison with state-of-the-art approaches.

Classification Technique	Top-1 Accuracy	F1 Score	AUC	Sensitivity	Specificity
SVM	92.34%	0.91	0.92	0.93	0.92
LR	91.60%	0.91	0.90	0.90	0.92
RF	93.40%	0.92	0.90	0.92	0.93
1DCNN (proposed)	97.15%	0.98	0.98	0.96	0.99

**Table 4.** Model quantization results.

Quantization	Top-1 Accuracy	Energy (pJ)	Model Size (bits)
2-bit/parameter	88.93%	7089	92,258
4-bit/parameter	88.98%	15,994	184,516
8-bit/parameter	93.00%	29,871	369,032
16-bit/parameter	96.55%	57,640	738,064
32-bit/parameter	97.03%	113,386	1,476,128
Baseline 1DCNN	97.15%	134,613	2,952,256

**Table 5.** SNN Accuracy and Energy Results.

Model	Top-1 Accuracy	Energy (pJ)
Baseline 1DCNN	97.15%	134,613
2-bit quantized 1DCNN	88.93%	7089
8-bit quantized 1DCNN	93.00%	29,871
SNN	93.33%	7282
